# Percutaneous Internal Ring Suturing (PIRS) for Inguinal Hernia Repair in Children—A Systematic Review

**DOI:** 10.3390/jcm15218579

**Published:** 2026-11-03

**Authors:** Dominika Biernaczyk, Marcin Polok

**Affiliations:** 1 Student Research Group of Pediatric Surgery and Urology, University of Zielona Góra, 65-417 Zielona Góra, Poland; 2 Department of Pediatric Surgery and Urology, University of Zielona Góra, 65-417 Zielona Góra, Poland

**Keywords:** PIRS, percutaneous internal ring suturing, pediatric inguinal hernia, laparoscopic hernia repair, recurrence, complications

## Abstract

**Background:** This study aimed to evaluate the effectiveness of percutaneous internal ring suturing (PIRS) for pediatric inguinal hernia. While previous reviews have addressed pediatric laparoscopic hernia repair more broadly, the present review specifically focuses on PIRS, with particular emphasis on recurrence rates. **Methods:** We searched PubMed, Web of Science, and Scopus for studies evaluating PIRS in children. The database searches were conducted between May 2025 and July 2026. Studies were eligible if they included at least 50 pediatric patients with inguinal hernia, reported a minimum follow-up of 6 months, and provided recurrence rates. Of 206 articles screened, 10 studies met the inclusion criteria; study characteristics and outcomes were summarized descriptively. Risk of bias was assessed using JBI tools and RoB 2, as appropriate. **Results:** The included studies comprised 1934 patients undergoing 2405 PIRS procedures. Overall, 80 complications (3.33%) were reported, including 39 recurrences (1.62%). Other complications included hematomas, iliac vessel punctures, granulomas, and transient hydroceles. One patient developed adhesive ileus with bowel strangulation, which required resection of a part of the ileum. Intraoperative assessment identified 190 additional bilateral hernias, increasing the rate of bilateral cases from 11.92% to 18.88%. Among the 1061 patients with available contralateral patent processus vaginalis (CPPV) data, CPPV was identified in 194 (18.28%). One case of complete androgen insensitivity syndrome (CAIS) was identified during PIRS. The weighted mean age reported across eight studies was 3.05 years. Mean operative time ranged from 8.48 to 26.5 min for unilateral and 18.0 to 33.9 min for bilateral procedures. **Conclusions:** PIRS appears to be an effective minimally invasive technique for pediatric inguinal hernia repair, with a low reported recurrence and complication rate. The possibility of closing the inguinal canal on the opposite side and assessing the gonads in girls during the procedure seems to provide an advantage over open techniques.

## 1. Introduction

Inguinal hernia (IH) is one of the most common surgical diseases in childhood. It occurs in 1–5% of children, rising sharply to over 30% in preterm infants and is 8–10 times more common in boys [1,2,3,4,5]. Concern for organ incarceration and necrosis necessitates surgical intervention in all children diagnosed with IH [6]. The most common treatment method used in children is the classic open method known as herniotomy. However, there are alternative laparoscopic surgical techniques, including the minimally invasive technique introduced by Patkowski in 2004 [7].

Surgery—PIRS (percutaneous internal ring suturing) is a laparoscopic technique that involves the percutaneous closure of a deep inguinal ring with a suture inserted via an injection needle through one 5 mm laparoscopic port located in the navel [7]. This technique is gradually gaining support in Europe and other continents, as evidenced by subsequent reports published in recent years [2,5,8,9,10,11,12,13,14,15,16,17,18]. The advantages of this surgical method are a minimal postoperative scar, a relatively simple surgical technique, and the possibility of assessing and treating the hernia on the opposite side. For girls, this technique also allows the uterus and appendage defects to be assessed [1].

Several systematic reviews have investigated the surgical treatment of inguinal hernia in children, including comparisons of laparoscopic and conventional open repair, as well as comparisons between different laparoscopic techniques [19,20]. These studies encompass a range of operative techniques and therefore substantial technical heterogeneity. In contrast, the present systematic review focuses specifically on percutaneous internal ring suturing (PIRS) and its effectiveness in children, with particular emphasis on recurrence rates, which remain an important outcome when assessing the durability of this technique. By limiting the analysis to a single laparoscopic technique, we aimed to reduce heterogeneity related to different operative approaches and provide a more focused assessment of the available evidence on PIRS.

## 2. Materials and Methods

**Figure 1:**
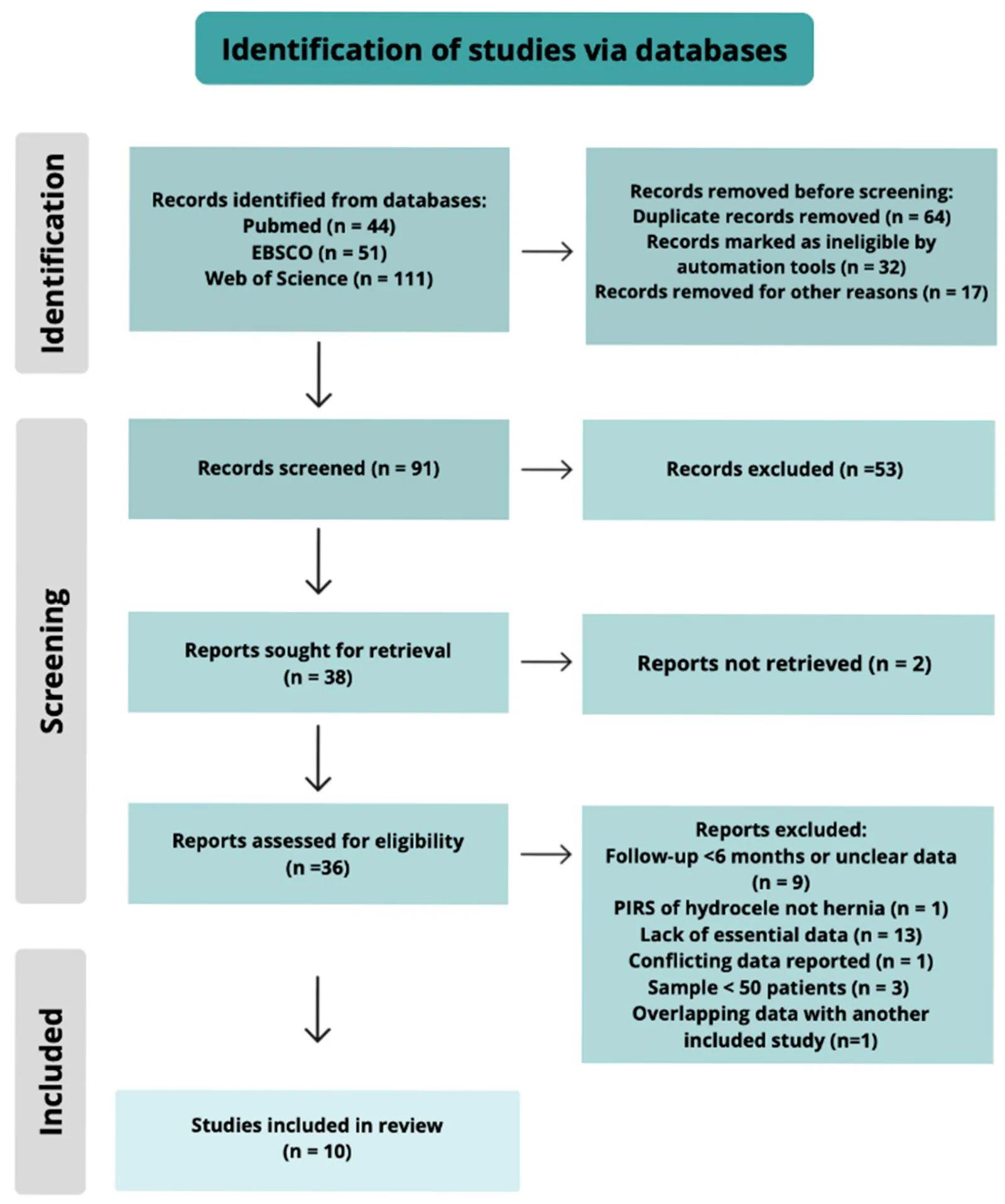
PRISMA flow diagram of the selection process for the studies included in the systematic review.

This systematic review was conducted in accordance with the PRISMA guidelines. The completed PRISMA 2020 checklist is provided in Supplementary Table S1. The review protocol was not registered in PROSPERO or any other systematic review registry; however, the review was conducted according to predefined eligibility criteria and a predefined search strategy established prior to the literature search. PubMed, Web of Science, and Scopus were searched from July 2025 to May 2026 for scientific works about laparoscopic surgery—PIRS in children using the following Boolean operators: “PIRS AND children AND hernia” and “percutaneous internal ring suturing AND hernia AND children”. The study selection process is illustrated in Figure 1 (PRISMA flow diagram).

During the preparation of this manuscript, the authors used ChatGPT (GPT-5.6 Luna; OpenAI, San Francisco, CA, USA) for English language editing and improving the clarity and readability of the text. No generative AI tools were used for data extraction, statistical analysis, interpretation of results, or formulation of the study conclusions. The authors reviewed and edited the AI-assisted output and take full responsibility for the content of this publication.

Ultimately, after analyzing 206 articles, 10 were included using the following criteria: (1) PIRS surgery as a treatment method; (2) material representing a minimum of 50 pediatric patients with inguinal hernia; (3) postoperative control of at least 6 months; and (4) recurrence rate given. The aim of the study was to assess the effectiveness of treatment. Results include data on complications and the number and rate of recurrence. 

The methodological quality of the included studies was independently assessed by two authors using the Joanna Briggs Institute (JBI) Critical Appraisal Checklists appropriate for the respective study designs. Cohort studies were assessed using the JBI Critical Appraisal Checklist for Cohort Studies, while case series were assessed using the JBI Critical Appraisal Checklist for Case Series. The risk of bias of the randomized controlled trial was independently assessed by the same two authors using the Cochrane Risk of Bias 2 (RoB 2) tool. The results of these assessments are presented in the Supplementary Tables S2–S4. 

The works covered various time periods and various countries in Europe: Poland, Turkey, Sweden, Croatia, the Netherlands, and Egypt. The oldest was published in 2006, and the largest number of works (8/10) were published between 2020 and 2026.

### Operating Technique

The technique of PIRS is described by its author as follows:

The PIRS procedure is performed under general endotracheal anesthesia with muscle relaxation, with the patient in the supine position. Pneumoperitoneum is established using an open technique by inserting a reusable 2.5 or 5 mm trocar through a transverse incision in the lower part of the umbilicus. The insufflation pressure is maintained at 8–10 mmHg, depending on the patient’s age. The trocar size depends on the diameter of the laparoscope. Either a 2.5 mm, 5-degree laparoscope or a 5 mm laparoscope with a 5- or 25-degree viewing angle may be used. The entire peritoneal cavity is inspected, and any hernia contents are reduced manually or with the aid of the laparoscope tip. All needle manipulations are performed under direct laparoscopic visualization, with the needle introduced and manipulated from outside the abdominal cavity. 

**Figure 2:**
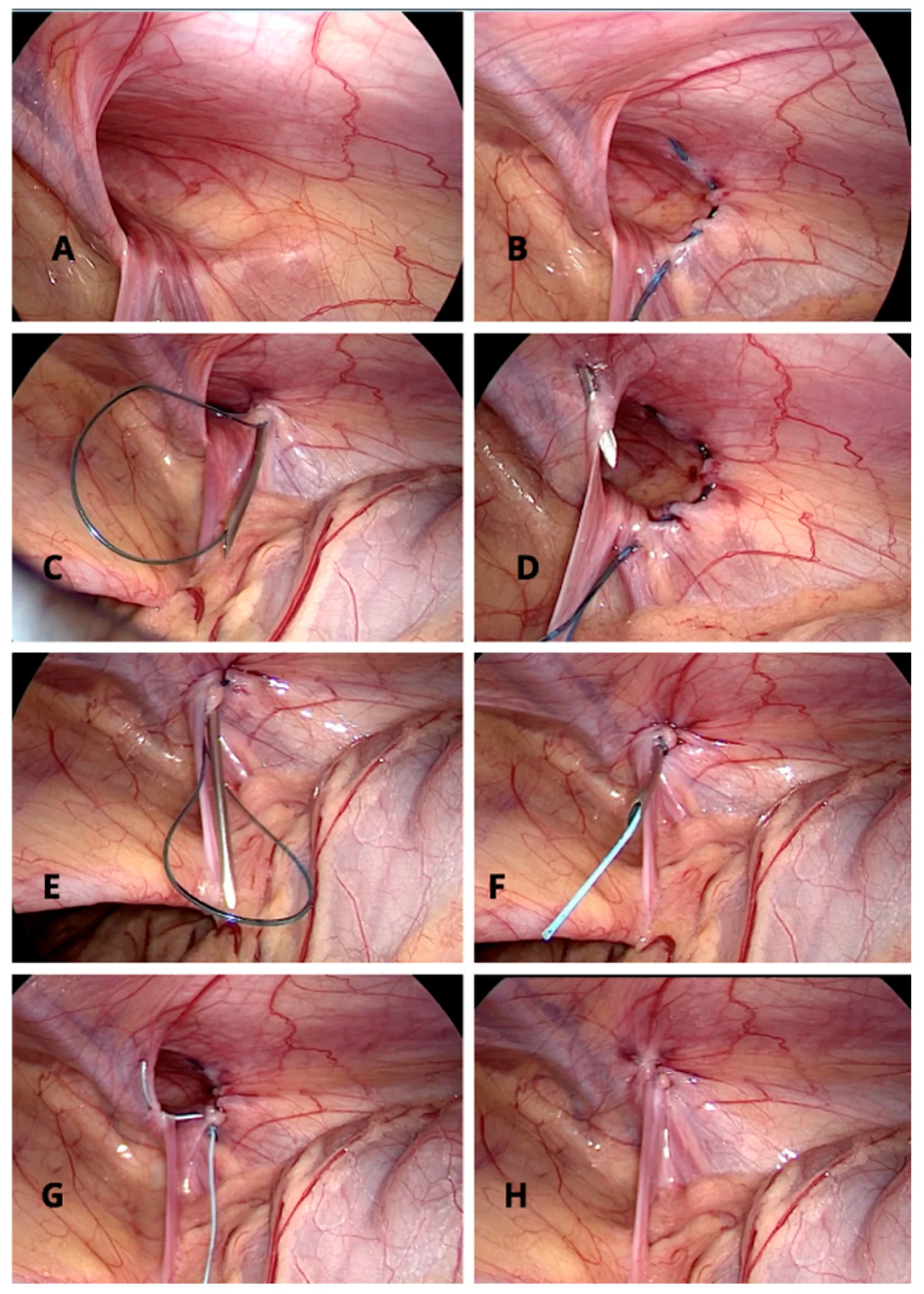
Main steps of the percutaneous internal ring suturing (PIRS) technique: (**A**) Identification of the patent internal inguinal ring under direct laparoscopic visualization. (**B**) Passage of the auxiliary suture around the first part of the internal inguinal ring to create a loop. (**C**) Formation of the auxiliary suture loop within the abdominal cavity. (**D**) Passage of the definitive suture around the remaining part of the internal inguinal ring. (**E**) Passage of the definitive suture with the auxiliary suture loop. (**F**) Withdrawal of the definitive suture from the needle lumen. (**G**) Withdrawal of the auxiliary suture loop, pulling the definitive suture around the internal inguinal ring and through the same skin puncture site. (**H**) Closure of the internal inguinal ring by tying the suture externally. All images were obtained at the Department of Pediatric Surgery and Urology, Mother and Child Health Centre, University Hospital in Zielona Góra, Poland.

To determine the needle puncture site, the position of the internal inguinal ring is assessed by pressing the inguinal region externally with the tip of a Pean forceps (Figure 2A). Under direct laparoscopic visualization, an 18-gauge injection needle containing a non-absorbable 2-0 monofilament suture is introduced through the abdominal wall into the peritoneal cavity. By manipulating the needle tip, the suture is passed beneath the peritoneum around one half of the internal inguinal ring, including a part of the ligament and adjacent tissue (Figure 2B). The suture is advanced through the lumen of the needle into the peritoneal cavity, forming a loop (Figure 2C). The needle is then withdrawn, leaving the suture loop inside the abdominal cavity. Outside the patient’s body, one end of the suture is introduced into the lumen of the needle again. The needle is passed through the same skin puncture site to encircle the remaining half of the internal inguinal ring, including the adjacent peritoneal and connective tissue (Figure 2D). To avoid injury to the vas deferens and testicular vessels, a small gap is left above these structures. The end of the suture is then passed through the lumen of the needle into the previously created loop, and the needle is withdrawn (Figure 2E,F). The auxiliary suture loop is subsequently pulled out of the abdominal cavity, carrying the definitive suture with it. This maneuver positions the definitive suture around the internal inguinal ring beneath the peritoneum, with both ends exiting through the same skin puncture site (Figure 2G). The ends of the definitive suture are tied outside the abdominal cavity, and the knot is buried subcutaneously to close the internal inguinal ring (Figure 2H).

If a patent contralateral internal inguinal ring is identified, it is closed during the same procedure, regardless of its diameter. The umbilical incision is closed with absorbable sutures and covered with a pressure dressing to prevent hematoma formation. The inguinal skin puncture site is left without a dressing [7].

## 3. Results

The results of the included studies are presented in Table 1. In total, 1934 patients were operated on, including 1260 (65.15%) boys and 674 (34.85%) girls. The age range of the children was 0.01 to 17 years. Among the seven studies that reported mean age, values ranged from 1.25 to 5.83 (weighted mean 3.05) years, calculated by weighting each study’s mean by its sample size. In two other studies, the authors provided only median ages, which were 2.17 and 13 years, respectively.

Preoperatively, a total of 1687 (89.08%) unilateral hernias and 264 (11.92%) bilateral hernias were found. The number of bilateral hernias intraoperatively increased to 454 (18.88%), which means that 190 patients had a “silent” contralateral hernia. Also, 41.85% of children operated on with bilateral hernias were left with an untreated hernia on the opposite side. Not only a “mute” hernia but also a contralateral patent processus vaginalis (CPPV) can be diagnosed intraoperatively. There were three studies where CPPV presence was described [1,6,11]. Out of 1061 patients, 194 (18.28%) were diagnosed with CPPV. 

Regarding operative characteristics, the procedures were performed using endoscopes with diameters of 2.5, 3, 3.5, and 5 mm. In two cases involving boys older than 15 years, a 10 mm 3D telescope was used [1]. Procedure duration was reported in seven studies, five of which provided separate data for unilateral and bilateral repairs. The reported mean duration ranged from 8.48 to 26.5 min for unilateral procedures and from 18.0 to 33.9 min for bilateral procedures. In addition, Danielson et al. reported a mean PIRS procedure duration of 26.54 min, while Eurlings et al. reported a mean duration of 38.2 min.

Overall, 80 complications (3.32%) were reported, comprising 39 recurrences (1.62% per procedure) and other complications accounting for 1.7%. As shown in Table 2, the included studies reported 39 recurrences in total (mean 2.02% per patient and 1.62% per procedure, ranging from 0 to 8.0%). In two studies from Turkey and Croatia, no recurrence was found. The highest percentage of recurrence was found in the work from Sweden—8.0%. According to Thomas et al., the recurrence rate was approximately three times higher following bilateral hernioplasty than unilateral hernioplasty, occurring in 3 patients (1.69%) versus 4 patients (0.61%), respectively. 

Suture material was reported in 8 of 10 studies (Table 3). Monofilament sutures were used in 1409 patients (71.14%), with recurrence rates of 1.81% per procedure and 2.20% per patient. Polyfilament sutures were used in 227 patients (11.73%), with corresponding recurrence rates of 2.48% and 3.08%, respectively. Suture material was not reported for the remaining 298 patients.

The authors reported that it was necessary to use an additional 5 mm trocar to manipulate bowels that obstructed the view in three cases (3.00%) in the study from Sweden. In the 2022 study by Wolak et al., additional tools were used in fourteen cases (5.67%). An additional 3.5 mm or 5.0 mm port was placed in the right lower abdominal quadrant for the grasper or dissector. During the procedures, operators used 16G, 18G, 20G, 21G, and 22G needles. The follow-up time ranged from 6 to 133.2 months.

The methodological quality of the included studies was assessed using the Joanna Briggs Institute (JBI) Critical Appraisal Checklist for Case Series and the JBI Critical Appraisal Checklist for Cohort Studies, as appropriate to the study design. The randomized controlled trial was assessed using the Cochrane Risk of Bias 2 (RoB 2) tool. Overall, the included cohort studies and case series showed generally acceptable methodological quality. The most common methodological limitations concerned the identification and management of potential confounding factors and, in some studies, incomplete or insufficiently addressed follow-up. Several cohort studies did not report specific strategies for controlling confounding factors, despite identifying potential differences between groups. Incomplete follow-up was reported in only one study, with reasons for loss to follow-up provided. The randomized controlled trial was assessed using the RoB 2 tool across five domains: bias arising from the randomization process, bias due to deviations from intended interventions, bias due to missing outcome data, bias in measurement of the outcome, and bias in selection of the reported result. The detailed results of the methodological quality and risk of bias assessments are presented in Supplementary Tables S2–S4.

## 4. Discussion

The laparoscopic PIRS technique is gradually gaining followers and is used in an increasing number of centers in Europe (Turkey [6,8,9,10], Sweden [11], the Czech Republic [14], Portugal [15], Hungary [2], and Finland [16]) and around the world (South Africa [17] and the USA [18]). However, there are some concerns about its use. In one publication from Turkey, the material covered only females, with the fear of damaging the vas deferens given as the reason in one of them [8]. In total, in all studies, the percentage of females was 34.85%, although it is known that inguinal hernia is 8–10 times more common in boys [1,2,3,4].

It seems that many centers do not use PIRS surgery due to the lack of experience in laparoscopy. However, the literature emphasizes that this technique is simple and fast in experienced hands [11,12,13,14]. The operation is performed with one trocar used only for optics. Only occasionally, in complicated cases, is it necessary to add an additional trocar. This technique does not require the skills of laparoscopic suturing, which clearly simplifies the operation compared to laparoscopic techniques where the hernial sac is tied transperitoneally with sutures inserted through the ports. PIRS also offers cosmetic benefits, including a single, small, umbilical scar that often becomes barely visible within a few months [1,7,8,9,12].

The studies included in our review do not provide sufficient data to assess the impact of suture type on recurrence rates. It has been hypothesized that polyfilament sutures, due to their tendency to induce a greater inflammatory response, may contribute to a reduction in recurrence rates. Among the studies analyzed in this review, only Kara et al. reported the use of polyfilament sutures, with a recurrence rate of 3.00%. Three studies did not specify the type of suture used, while the remaining studies reported the use of monofilament sutures. Therefore, it cannot be concluded that either polyfilament or monofilament sutures are associated with lower recurrence rates. Notably, Thomas et al., Akin et al., and Erginel et al. reported recurrence rates of 0% or 0.98% despite the use of monofilament sutures. However, the lack of detailed information on suture characteristics limits the interpretation of these findings. Furthermore, some centers used sutures of different thicknesses, making it difficult to assess the relationship between suture thickness and recurrence rates. Other factors may also have contributed to the observed differences in recurrence rates.

Opponents of the method indicate a greater recurrence rate compared to the classical method. This systematic review showed a 2.02% recurrence rate per patient and 1.62% per procedure, which is comparable to the recurrence rate following the classical method reported in the literature (0.4–2.4%) [3,13,21]. Out of 1934 patients, only one experienced a serious complication involving bowel obstruction and subsequent bowel resection. Although serious complications were rare in the included studies, this finding should be interpreted with caution when assessing the overall safety of the technique.

The mechanisms underlying recurrence after PIRS may involve both technical and anatomical factors [22,23]. In one of the included studies, Saad et al. reported a recurrence attributed to a slipped ligature, which was managed by dissecting the peritoneum at the level of the internal inguinal ring (IIR) and narrowing the ring with additional sutures [23]. This case highlights the importance of secure suture placement and effective closure of the IIR. The authors also emphasized the potential relevance of IIR diameter in individualizing surgical management, suggesting that intraoperative assessment of ring size could help determine whether additional tissue repair is warranted [23]. Anatomical variability in IIR diameter may contribute to differences in surgical outcomes, including recurrence; however, further studies are needed to establish whether ring size can reliably guide surgical decision-making and reduce recurrence after PIRS.

Moreover, this method seems to be beneficial for patients. The importance of assessing and simultaneously closing the second inguinal canal during the PIRS procedure should be noted. The most common cause of developing an inguinal hernia in children is a patent processus vaginalis or canal of Nuck. The incidence of contralateral PPV at operation has been estimated to be 21–47%, as deduced from laparoscopic studies [11]. Hence, another great advantage of PIRS is the possibility of assessing the other inguinal canal in terms of CPPV presence. There is no clear statement indicating whether to close CPPV or not [24,25,26]. According to studies by Koivusalo and Maddox, 11% to 25% of children with CPPV may develop an inguinal hernia [20,27]. To reduce the risk of developing inguinal hernias, Danielson opted to close PPV during PIRS, acknowledging that long-term studies on the outcomes of asymptomatic PPV closure remain limited. The data collected in the systematic review showed that 190 additional contralateral hernias were identified intraoperatively. These represented 9.82% of the total number of patients and 7.90% of all hernias included in the review. This means that nearly 190 patients were spared from undergoing repeat surgical procedures, resulting in a reduction in patient stress, pain, time, and associated costs. Some of them may have experienced recurrence; such details were not reported.

Another important advantage of laparoscopic surgery is the macroscopic evaluation of gonads in girls [1,4]. Interestingly, one of the initial applications of laparoscopy in pediatric inguinal hernia repair, introduced in the early 1990s, was the assessment of the contralateral inguinal region due to the high incidence of bilateral inguinal hernias in children [28]. With the subsequent development of laparoscopic techniques, their diagnostic potential extended beyond the detection of contralateral hernias, allowing direct visualization of the gonads and other intra-abdominal structures. The literature has emphasized various anatomical abnormalities that may be identified incidentally during laparoscopy in girls, including a unicornuate uterus, ovotestis, and fallopian tube abnormalities [1,5,10]. In the study by Wolak et al., one patient was diagnosed with complete androgen insensitivity syndrome (CAIS), while intestinal abnormalities were identified in six cases, including intestinal malrotation in five children and Meckel’s diverticulum in one [1]. Thus, in addition to its therapeutic role, laparoscopy may provide valuable diagnostic information that would not be available during conventional open repair.

Laparoscopic surgery also offers the opportunity to perform umbilical reconstruction in children with an umbilical hernia. In Wolak’s 2022 study, preoperative assessment identified ten cases of umbilical hernia, while a further ten children were found to have a widened umbilical ring requiring reconstruction [1].

Beyond these additional procedural possibilities, recent prospective comparative evidence has also highlighted potential perioperative benefits of PIRS. Eurlings et al. reported significantly shorter anesthesia times with PIRS compared with LIHR (laparoscopic inguinal hernia repair), whereas the difference in net surgical time did not reach statistical significance, despite a trend toward shorter operative times with PIRS [29]. These findings may support the efficiency of PIRS, although further studies with standardized definitions of operative time are needed to confirm this potential advantage.

Some limitations of the present review should nevertheless be considered. One included study reported only the mean follow-up rather than the minimum follow-up duration. However, the mean follow-up of 70.6 months made it highly likely that the study met the predefined minimum follow-up criterion of 6 months. The relatively small number of included studies, predominantly retrospective study designs, differences in study follow-up duration, and incomplete reporting of some outcomes may also limit the strength and comparability of the available evidence. In addition, the literature search and initial data extraction were performed by one author, while the extracted data and study selection were subsequently checked by a second author.

High-quality studies involving large patient cohorts are needed to strengthen the current evidence. Future research should include a follow-up period of at least six months, report recurrence rates per patient and per procedure, and specify the suture materials used. Intraoperative findings, including contralateral patent processus vaginalis (CPPV) and occult contralateral hernias, should also be consistently documented. Reporting outcomes stratified by age group would help determine whether age influences recurrence risk and other clinical outcomes. Further investigation into the factors contributing to recurrence after PIRS is also warranted.

## 5. Conclusions

The available evidence suggests that PIRS is an effective treatment option for inguinal hernia in children, with a low reported recurrence rate and a low overall complication rate. The laparoscopic approach also allows intraoperative assessment of the contralateral internal inguinal ring and may offer cosmetic benefits due to the use of single umbilical access. However, the interpretation of recurrence rates is limited by differences in outcome reporting and follow-up duration across studies, as well as the predominantly retrospective nature of the available evidence. Further prospective studies with standardized outcome definitions, consistent reporting of recurrence rates and complications, and long-term follow-up are needed to better establish the effectiveness and safety of PIRS.

## Figures and Tables

**Table 1:** Characteristics of the studies included in the systematic review.

First Author	Study Location	Year of Publication	Study Duration	Number of Patients	Gender		Age [Years]		
					M No. (%)	F No. (%)	Median	Mean	Age Range
Patkowski	Wroclaw, Poland	2006	2004–2005	106	86 (81.13%)	20 (18.87%)	No data	3.4	0.08 *–14.5
Erginel	Istanbul, Turkey	2016	2010–2014	148	0 (0%)	100 (100%)	No data	5.83	0.08 *–16.0
Kara	Ankara, Turkey	2022	2014–2019	227	144 (63.44%)	83 (36.56%	No data	4.56	0.25 *–17.0
Akin	Istanbul, Turkey	2022	2012–2017	164	0 (0%)	164 (100%)	No data	5.3	0.08 *–17.0
Danielson	Uppsala, Sweden	2021	2014–2017	100	82 (82%)	18 (18%)	2.17	No data	No data
Wolak	Kielce, Poland	2022	2008–2016	247	145 (52.5%)	131 (47.46%)	3.51	4.83	0.25 *–17.0
Pogorelić	Split, Croatia	2022	2015–2021	51	37 (72.55%)	14 (27.45%)	13	No data	10.0–17.0
Thomas	Istanbul, Turkey	2024	2013–2021	714	520 (72.82%)	194 (27.17%)	1.65	1.66	0.01 *–10.08
Eurlings	Maastricht, the Netherlands and Wroclaw, Poland	2026	No data	124	90 (72.58%)	34 (27.42%)	No data	2.7	1.4–3.5
Saad	Alexandria, Egypt	2025	2022–2024	53	53 (100%)	0 (0%)	No data	1.25	0.5–5.0
				1934	1260 (65.15%)	674 (34.85%)			

* Values converted from days/months to years: 28 days = 0.08 years; 90 days = 0.25 years; 0.1 months = 0.01 years.

**Table 2:** Results.

First Author	Preoperative Diagnosis		Final Diagnosis		Total Number of Operated Hernias	CPPV	Suture Material	Average Operating Time [Minutes]	Late Complications (Recurrence Per Patient) (%)	Late Complications (Recurrence Per Procedure) (%)	Other Complications	Follow-Up [Months]
	Unilateral	Bilateral (%)	Unilateral	Bilateral (%)								
Patkowski	94	12 (11.32%)	72	34 (32.08%)	140	No data	2/0 non-absorbable monofilament	Unilateral 19.36 Bilateral 24	3 (2.83%)	3 (2.14%)	iliac vessel punctures (*n* = 3), adhesion ileus with bowel strangulation (*n* = 1), transient hydroceles (*n* = 5)	18–29
Erginel	117	31 (20.95%)	91	57 (38.51%)	205	No data	2/0 non-absorbable monofilament	No data	0 (0.00%)	0 (0.00%)	Hematoma (*n* = 2), granuloma (*n* = 1)	30–73
Kara	196	19 (8.84%)	148	67 (29.94%)	282	No data	2/0 or 3/0 non-absorbable braided	No data	7 (3.08%)	7 (2.48%)	Hematoma (*n* = 6), stitch abscesses (*n* = 3)	6–69
Akin	126	38 (23.17%)	99	65 (39.6%)	229	No data	2–0/3–0 monofilament	No data	1 (0.61%)	1 (0.44%)	Hematoma (*n* = 4), Granuloma (*n* = 1)	6–56
Danielson	82	18 (18%)	65	35 (35.00%)	135	17 (17%)	3/0 polypropylene	26.5	8 (8.00%)	8 (5.39%)	0	6
Wolak	247	29 (11.70%)	197	79 (28.62%)	355	53 (19.2%)	No data	Unilateral 26.5 Bilateral 32.7	10 (4.04%)	10 (2.82%)	No data	70, 6 average
Pogorelić	45	6 (13.30%)	45	6 (13.30%)	57	No data	No data	Unilateral 10 Bilateral 18	0 (0.00%)	0 (0,00%)	Inferior epigastric vein injury (*n* = 1)	average 44, minimum 6
Thomas	660	54 (7.60%)	660	54 (7.60%)	768	124 (17.4%)	2/0 non-absorbable monofilament	Unilateral 13.6 Bilateral 33.9	7 (0.98%)	7 (0.91%)	Omental evisceration (*n* = 1), hematoma (*n* = 1), iliac vessel puncture (*n* = 5)	36–133, 2
Eurlings	74	50 (12.32%	74	50 (12.32%)	174	No data	monofilament polydioxanone suture (PDS)	38.2	2 (1.61%)	2 (1.15%)	Suture fistulas (*n* = 2) granuloma (*n* = 1) fibroma (*n* = 1) umbilical infection (*n* = 1) testicular atrophy (*n* = 1)	12
Saad	46	7 (13.2%)	46	7 (13.2%)	60	No data	2/0 monofilament suture	Unilateral 8.48 mean 21.42	1 (1.89%)	1 (1.66%)	Edema (*n* = 1)	12
	1687	264 (11.92%)		454 (18.88%)	2405				39 (2.02%)	39 (1.62%)	41	

Abbreviations: CPPV—contralateral patent processus vaginalis.

**Table 3:** The relationship between the type of sutures and the recurrence rate.

Title 1	Monofilament	Polyfilament	Not Reported
Number of patients	1409	227	298
Number of procedures	1711	282	412
Percentage of procedures	71.14%	11.73%	17.13%
Number of recurrences	31	7	N/A
Recurrence rate per procedure	1.81%	2.48%	N/A
Recurrence rate per patient	2.2%	3.08%	N/A

N/A—not applicable.

## Data Availability

The data analyzed in this study were obtained from the published studies cited in the manuscript. No additional datasets were generated.

## Supplementary Materials

The following supporting information can be downloaded at https://www.mdpi.com/article/10.3390/jcm15218579/s1. Table S1: PRISMA 2020 checklist [30]; Table S2: Risk-of-bias assessment of the included case series using the Joanna Briggs Institute (JBI) Critical Appraisal Checklist for Case Series [31]; Table S3: Risk-of-bias assessment of the included cohort studies using the Joanna Briggs Institute (JBI) Critical Appraisal Checklist for Cohort Studies [32]; Table S4: Risk-of-bias assessment of the included randomized controlled trial using the Cochrane Risk of Bias 2 (RoB 2) tool [33].

## Abbreviations

The following abbreviations are used in this manuscript:

PIRS	Percutaneous internal ring suturing
CPPV	Contralateral patent processus vaginalis
N/A	Not applicable
LIHR	Laparoscopic inguinal hernia repair
CAIS	Complete androgen insensitivity syndrome
IIR	Internal inguinal ring
