# Supplementary material for: Percutaneous Internal Ring Suturing (PIRS) for Inguinal Hernia Repair in Children—A Systematic Review
Source: J Clin Med. 2026 Nov 3;15(21):8579. doi: 10.3390/jcm15218579 (PMC13764187; doi:10.3390/jcm15218579)
Supplement: Supplementary file 1 [file jcm-15-08579-s001.zip › Supplementary Tables S2, S3, S4 Risk of Bias Assessment - PIRS.pdf]

Supplementary Table S2. Risk of bias assessment of the included case series using the Joanna Briggs Institute (JBI) Critical Appraisal Checklist for Case Series.

**Study**	**Q1**	**Q2**	**Q3**	**Q4**	**Q5**	**Q6**	**Q7**	**Q8**	**Q9**	**Q10**	**Total**
	Were there clear criteria for inclusion in the case series?	Was the condition measured in a standard, reliable way for all participants included in the case series?	Were valid methods used for identification of the condition for all participants included in the case series?	Did the case series have consecutive inclusion of participants?	Did the case series have complete inclusion of participants?	Was there clear reporting of the demographics of the participants in the study?	Was there clear reporting of clinical information of the participants?	Were the outcomes or follow up results of cases clearly reported?	Was there clear reporting of the presenting site(s)/clinic(s) demographic information?	Was statistical analysis appropriate?	Out of 10
Patkowski et al., 2006	Yes	Yes	Yes	Unclear	Unclear	Yes	Yes	Yes	Yes	N/A	7
Erginel et al. 2016	Yes	Yes	Yes	Unclear	Unclear	Yes	Yes	Yes	Yes	N/A	7

Supplementary Table S3. Risk of bias assessment of the included cohort studies using the Joanna Briggs Institute (JBI) Critical Appraisal Checklist for Cohort Studies.

Study	Q1	Q2	Q3	Q4	Q5	Q6	Q7	Q8	Q9	Q10	Q11	Total
	Were the two groups similar and recruited from the same population?	Were the exposures measured similarly to assign people to both the exposed and unexposed groups?	Was the exposure measured in a valid and reliable way?	Were confounding factors identified?	Were strategies to deal with confounding factors stated?	Were the groups/participants free of the outcome at the start of the study (or at the moment of the exposure)?	Were the outcomes measured in a valid and reliable way?	Was the follow-up time reported and sufficient to be long enough for outcomes to occur?	Was follow-up complete, and if not, were the reasons to loss to follow up described and explored?	Were strategies to address incomplete follow-up utilized?	Was appropriate statistical analysis used?	Out of 11
Kara et al. 2022	Yes	Yes	Yes	No	No	Yes	Yes	Yes	Yes	N/A	Yes	8
Akin et al. 2022	Yes	Yes	Yes	No	No	Yes	Yes	Yes	Yes	N/A	Yes	8
Danielson et al. 2021	Yes	Yes	Yes	Yes	No	Yes	Yes	Yes	Yes	Yes	Yes	11
Wolak et al. 2022	Yes	Yes	Yes	Yes	No	Yes	Yes	Yes	Yes	N/A	Yes	9
Pogorelić et al. 2022	N/A	N/A	Yes	No	N/A	Yes	Yes	Yes	Yes	N/A	Yes	6
Thomas et al. 2024	Yes	Yes	Yes	Yes	No	Yes	Yes	Yes	Yes	N/A	Yes	9
Eurling et al. 2026	No	Yes	Yes	Yes	Yes	Yes	Yes	Yes	Yes	No	Yes	9

Supplementary Table S4. Risk of bias assessment of the included randomized controlled trial using the Cochrane Risk of Bias 2 (RoB 2) tool.

Study	D1	D2	D3	D4	D5	Overall risk of bias
	Risk of bias arising from the randomization process	Risk of bias due to deviations from the intended interventions (*effect of assignment to intervention*)	Missing outcome data	Risk of bias in measurement of the outcome	Risk of bias in selection of the reported result	
Saad et al. 2026	Low	Low	Low	Low	Some concerns	Some concerns
